# Employee well-being and innovativeness: A multi-level conceptual framework based on citation network analysis and data mining techniques

**DOI:** 10.1371/journal.pone.0280005

**Published:** 2023-01-06

**Authors:** Yousif Elsamani, Cristian Mejia, Yuya Kajikawa

**Affiliations:** 1 Department of Innovation Science, School of Environment & Society, Tokyo Institute of Technology, Tokyo, Japan; 2 Institute for Future Initiatives, The University of Tokyo, Tokyo, Japan; The Islamia University of Bahawalpur Pakistan, PAKISTAN

## Abstract

This study proposes a multilevel conceptual framework for a deeper understanding of the relationship between employee well-being and innovativeness. We overview 49 years of well-being research [1972–2021] and 54 years of research on innovativeness [1967–2021] to uncover 24 dominant themes in well-being and ten primary topics in innovativeness research. Citation network analysis and text semantic similarity were used to develop a conceptual framework featuring 21 components and three levels: individual, organizational, and market. These components consist of constructs, domains, and factors that can influence or be influenced by employee well-being and innovativeness either directly or indirectly. This is the first study to use citation network analysis and data mining techniques to investigate the relationship between employee well-being and innovativeness. This novel framework can aid organizations in identifying more holistic and efficient strategies for fostering innovativeness and enhancing the well-being of their workforce. It can also assist in developing new theories and serve as a roadmap for future research. We discuss the research limitations and theoretical and practical implications and propose three research themes that future studies may address.

## Introduction

The world and business environment are changing rapidly at unprecedented speeds and scales in this digital era. In such an environment, innovation is critical; thus businesses continue to rely on their employees to find innovative solutions to never-ending challenges and problems [1, 2]. In addition, organizations must innovate to progress, compete, and differentiate themselves in the marketplace [3, 4]. Innovation is a process that begins with problem recognition and triggers an idea that needs to be promoted and implemented to realize its benefits. The successful execution of new ideas takes place via employees who, alone or in groups, work to turn them into innovations [4]. Therefore, it is no surprise that innovative workers are the most valuable assets in today’s organizations, and the question of how to enhance and sustain employees’ innovativeness is a core issue with which researchers and managers grapple. However, unlike researchers in human resource management and psychology, innovation scholars have paid little attention to well-being and its relationship with innovativeness [5]. Understanding the link between employee well-being and innovativeness is critical for determining how innovative employees can and should be supported and how we can improve the overall performance and resilience of an organization to meet future challenges and capitalize on opportunities.

Numerous studies have investigated well-being and innovativeness. Using”well-being” as a search query on Web of Science database, for example, results in thousands of articles. However, to make fruitful progress, we need to comprehend what is known so far and build new studies on this topic. Considering the enormous number of publications on well-being and innovativeness, it is challenging to achieve this using traditional and manual review methods; thus, bibliometric methods offer promising approaches that can help provide more comprehensive reviews and aid in understanding the relationship between constructs.

Previous researchers have conducted bibliometric studies to assess well-being. For example, Dominko conducted research on subjective well-being among the elderly [6] and Sánchez examined the well-being of entrepreneurs [7]. Another example is the study by Houlden et al., who investigated the relationship between greenspace biodiversity and human health and well-being [8]. They claimed that their research supported the association between health and floral biodiversity, especially subjective well-being and self-reported health. Jeter et al. investigated yoga as a health intervention in clinical populations using bibliometrics methods [9], and Ali et al. analyzed open-access articles on well-being [10]. Hernández conducted two studies, one of which looked at children’s well-being in the school context [11], and the other at university students’ well-being [12]. Similarly, researchers have attempted to provide an overview of the research on innovativeness using bibliometric methods. For example, Marchiori et al. conducted a review study using data downloaded from the Web of Science (2,523 articles) for publications from the time data collection began until 2018, selecting only the management and business categories to identify several topics that could be further investigated in the future [13]. Another study focusing only on the management and business categories considered publications from 1945 to 2012 [14]. Their co-citation analysis was performed only on 100 articles, which they referred to as "the most cited papers." Innovativeness and innovation measures have also been investigated in one study. The authors attempted to identify and classify the primary measures used by what they refer to as a "high-impact or recent" papers published over a ten-year period. Likewise, Dambiski et al. reviewed innovativeness measures over a 10-year period and proposed a new classification of organizations considering their inputs, capabilities, and outputs [15].

Hannele et al. investigated the relationship between employee well-being and innovativeness from the perspective of job demands and resources; they argue that burnout and engagement mediate the effects of resources and demands on innovativeness [16]. Unlike work engagement, burnout is viewed as a barrier to inventiveness. These findings were supported by those of Honkaniemi *et al*.*—and one* of their main findings suggests that a high level of well-being is linked to a high level of innovativeness and vice versa [17]. In sum, the scope of the existing bibliometric studies on well-being and innovativeness and the relationship between them tend to have a narrow focus because they usually consider one or a few constructs. Moreover, they are not comprehensive because they tend to focus only on a specific period or categories (e.g., management and business). Thus, these studies have failed to capture and reflect a more thorough and detailed illustration of the relationship between employee well-being and innovativeness and have neglected the dynamics between their related constructs and domains.

This study utilized bibliometric methods as the first step to identify and group (in clusters) the most relevant and high-quality (most cited) articles into research topics for each research field separately. It then uses a data mining technique (semantic similarity) to identify the potential links between these research topics. Such methods have been utilized and their effectiveness validated; for example, in determining links between social and technological issues (e.g., robotics and gerontology) [18]. Considering the large number of articles, however, searching manually (e.g., reading through articles) is challenging and time-consuming. Nevertheless, this study provides a comprehensive review of two topics (employee well-being and innovativeness) utilizing all available publications in the Web of Science database (up to November 2021). It then investigates the relationship between these two topics and their related constructs and domains at three levels: individual, organizational, and market. Furthermore, we propose a conceptual framework to deepen our understanding of the relationship. The framework can help organizations in their endeavors to find more integrated and effective ways to support their employees’ well-being and innovativeness, which can enhance organizational innovativeness and performance. Before reviewing the findings of our analysis, we provide a brief historical overview of research on innovativeness and well-being emphasizing scaling techniques and methodologies.

## Literature review

### Well-being and its measurement

Bradburn’s 1969 research, in which he defines happiness in terms of emotional experiences, is one of the earliest discussions on well-being. Specifically, he hypothesizes that people are happy when they have more positive than negative affect [19]. This perspective emphasizes pleasurable emotional encounters, which means that people are happier when they have enjoyable emotional experiences but fell less joyful during negative emotional encounters. However, in 1977, Coan discussed a new perspective of well-being, defining it in terms of external criteria and as a state to which an individual aspires to reach by achieving some desirable quality [20]. In 1978, Shin and Johnson added subjectivity to the definition of happiness, introducing a new viewpoint that happiness is a global evaluation of an individual’s quality of life based on the person’s criteria [21]. This subjective perspective has gained much traction since it was inspired by the question of what causes people view their lives positively [22]. Another central point of debate in the extant literature is the definition of mental health. In 1989 and 1993, Ryff and Waterman argued that mental health should be viewed as the presence of wellness rather than the absence of disease. In 1989, Ryff discussed five well-being constructs—autonomy, environmental mastery, positive relationships with others, purpose in life, potential realization, and self-acceptance [23]—and introduced the concept of psychological well-being (PWB) as a critical marker of good mental health. Waterman added subjectivity to this concept by claiming that happiness results from personal fulfillment and expressiveness [24]. Summing up all previous discussions, in 1997, Diener and Such concluded that subjective well-being comprises three interconnected components: life satisfaction, pleasant affect, and unpleasant affect, where life satisfaction refers to an individual’s cognitive sense of satisfaction with his life [25]. In 2015, Steptoe built on the concept of life satisfaction, claiming that it has a solid link to age. Andrew discussed three aspects of subjective well-being: evaluative well-being (life satisfaction), hedonic well-being (happiness, sadness, anger, stress, and pain), and eudemonic well-being (sense of purpose and meaning in life) [26].

Another vital contribution to the well-being literature is the research conducted by Keyes in 2002, 2005, and 2007, in which he introduced his complete state model of mental health [27–29]. Keyes’ research supports Ryff and Waterman’s claims that mental health and mental illness are two distinct but related dimensions [28]. He classified people as "flourishing" when they meet his mental health criteria and "languishing" when not. Ryff and Singer (2011) discussed the eudemonic vision of psychological well-being and provided empirical evidence of a biological correlation in the cardiovascular, neuroendocrine, and immune systems [30]. Building on the flourishing and languishing concept, Seligman proposed PERMA (positive emotion, engagement, relationships, meaning, and accomplishment) as building blocks for a flourishing life in the same year [31]. However, contrary to this flow and progress in well-being research, some researchers believe that most previous research has been overly focused on discussing well-being constructs and domains rather than defining well-being itself. This led Rachel to propose a dynamic vision of well-being in 2012 that defines well-being as the balance between an individual’s resource pool and challenges [32]. Another construct of well-being is capability well-being, defined in terms of what an individual can do and be in their life. This approach is essential for public policymakers and economic evaluations because it focuses on gathering information from the general public about important capabilities (stability, attachment, achievement, autonomy, and enjoyment) [33]. More recently, in 2020, a group of researchers gathered to discuss and propose a global understanding of well-being, which included hedonic and eudemonic facets of well-being, social well-being, and the role of culture, community, nature, and governance [34]. The importance of the relational dimension, which considers how social relationships enable people to act meaningfully in pursuit of what they consider happiness, was also discussed [35]. This approach is critical when examining well-being from an ecosystem perspective. Taking fisheries as an example, a three-dimensional well-being approach (material, relational, and cognitive) can provide valuable insights into how sustainable fishery policies can be developed [36].

On the other hand, apart from social attributes (e.g., education, health, political voice, social networks, and connections), the objective perspective of well-being defines it in terms of quality of life indicators, such as material resources (e.g., income, food, housing), [37]. The financial domain is related to objective well-being. In this domain, there are two related but separate constructs: stress related to the management of money today (everyday money management stress), and a sense of security in one’s financial future (expected future financial security) [38].

With the growing use of the Internet and social media, some researchers have looked at how these technological aspects may affect well-being. Vanden discussed the concept of "digital well-being" in 2021, which he defined as our inability to balance our experiences and relationships with mobile technology [39]. The inconsistency in previous research findings and the need for more rigorous methodologies to capture such a complex construct drove his research. However, as this article pays more attention to employees’ well-being, we were more interested in studies discussing workplace well-being. One of the most comprehensive frameworks related to employee well-being was developed by Kathryn in 2009. It discusses three main constructs of employee mental health (subjective well-being, workplace well-being, and psychological well-being) [40]. Researchers also recognize the dynamic nature of this construct, noting that employee well-being is not fixed and that whether it will remain stable in the future depends on various factors. For example, the employee’s age and whether he/she changes jobs can have a significant impact [41]. Researchers have introduced different constructs of well-being. Table 1 shows the major constructs of well-being and their definitions in the literature.

**Table 1:** Main constructs of well-being and their definitions from the literature.

Well-being constructs	Description from the literature	Study
Psychological	Psychological well-being (PWB):	Ryff, 1989 [23].
	Self-acceptance, Positive relations with others, Autonomy, Environmental mastery, Purpose in life, Self-growth.	
Subjective	Subjective well-being: people’s personal evaluations of their lives, especially:	Diener et al., 1997 [25].
	Life satisfaction (a cognitive evaluation).	
	Happiness (a positive emotional state).	
	Unhappiness (a negative emotional state).	
	Three aspects of subjective well-being: the link to age	Steptoe et al., 2015 [26].
	Evaluative well-being (or life satisfaction).	
	Hedonic well-being (happiness, sadness, anger, stress, and pain).	
	Eudemonic well-being (sense of purpose and meaning in life).	
Objective	This construct is defined by a person’s actual possessions. Housing and income, as well as intangible attributes such as education, health, and social connections, are used to define objective well-being.	Western and Tomaszewski, 2016 [37].
Capability	Defines well-being in terms of what an individual can ‘do’ and ‘be’ in their lives.	Sen, 1999 [33].
Financial	Financial well-being as two related but separate constructs:	Netemeyer et al., 2018 [38].
	(1) The stress of today’s financial management	
	(2) A feeling of assurance about one’s financial future	
Social	The importance of the relational dimension, which considers the extent to which social relationships enable the person to act meaningfully in pursuit of what they regard as well-being.	Umberson and Montez, 2010 [35].
Digital	Digital well-being is a subjective individual experience of optimal balance between the benefits and drawbacks obtained from mobile connectivity.	Vanden Abeele, 2021 [39].

Well-being measurements can be used at an individual or national level. Because economic measures such as gross domestic product (GDP) are insufficient for assessing the population’s quality of life, subjective social measures such as the Personal Well-being Index (PWI) are needed [42]. Scales used to measure national well-being in New Zealand [43] and Singapore [44] are examples of the PWI.

At the individual level, scales can vary depending on the circumstances being measured. As a result, the way the questionnaire or scale items are constructed differs. For example, the Post-Trans Questionnaire is a subjective well-being measure (SWB) developed explicitly for post-natal mothers with type 1 diabetes [45]. Other assessments are tailored to specific age groups, such as children [46] or adolescents [47]. One of the earliest scales to measure and evaluate children’s well-being is the scale developed by Harter (1982), which includes questions that probe students’ attitudes toward schools and academic aptitude [48]. Most children and adolescents’ well-being is discussed in the school context [49], and adults generally determine the conceptual parameters and methods used to evaluate their well-being. However, it is also essential to understand children’s perspectives on well-being [50]. Some scales are designed to assess only one construct of well-being, such as psychological [19], subjective [51], and capability well-being [52]. Others are occupational-oriented scales designed to assess the well-being of people who work in a specific sector, such as teachers [53], doctors [54], nurses [55], or more broadly, such as general employees [56, 57].

There are two major approaches in the literature for developing scales to measure employee well-being. The first approach is factor analysis (FA), which uses statistical methods to analyze correlations among primary scale items to determine which items should be included in the final scale [58]. The second is impact analysis (IA), which uses evaluation criteria to calculate an "impact" score for each item [59]. Juniper compared the two approaches and discussed the development of the Work and Well-Being Assessment (WWBA) scale [56]. Rabindra et al. noted the lack of a proper theoretical model for employee well-being in 2019 and attempted to develop a new scale using CFA [57]. The scale was developed using 62 items derived from interviews with academics and HR professionals and subsequently validated using exploratory factor analysis (EFA), which resulted in the retention of 31 items. Because the EFA revealed that the scale items covered social, psychological, subjective, and workplace well-being aspects, this scale can be considered an extensive scale, which is in line with Kathryn’s framework proposed in 2009 [40].

### Innovativeness and its measurement

Between 1970 and 1978, approximately 25 articles discussed the relationship between new product adoption, consumer innovativeness, and individual innovativeness (innate innovativeness), with contradictory empirical results suggesting a positive, negative, or no relationship between them [60]. These contradictory results suggest that a model of trait behavioral processes is needed. In 1978, Midgley wrote, "*Without a model of the processes intervening between trait and behavior*, *we are in no position to ascribe any meaning to empirical correlations*" claiming that such a model was crucial for advancing the field. Since then, numerous studies have been conducted to develop such models and define and measure innovativeness and its related constructs [60]. Twenty five years after Medgley’s statement, Goldsmith identified three ways to conceptualize innovativeness—behavioral, global trait, and domain-specific—and three ways to measure innovativeness: global traits, consumer innovativeness, and behavioral [61].

Employee innovativeness, commonly referred to in the literature as "innovative behavior," is a complex, highly context-specific, and problem-specific concept. Therefore, we must be cautious not to be overly mechanistic about this concept and to think only in terms of its antecedents and consequences [16]. Innovative behavior is triggered by first recognizing problems, after which novel ideas or solutions are generated. The resulting ideas must then be nurtured and encouraged (idea promotion) in order to be realized [4, 62, 63]. Individuals are likely to engage in any combination of the aforementioned behaviors [4]. We reach innovation when we can produce benefits by realizing new ideas [64]. To obtain the most out of the newly generated ideas, we need to consider improving this multi-stage process. Accordingly, researchers have debated this issue and proposed several factors as predictors of innovativeness. Some of the methods to improve this process are increasing job resources and decreasing demands [62], giving employees autonomy and some control over how they perform their tasks [65], and promoting a culture that supports innovativeness [66]. Leadership and some leader characteristics, such as authenticity, can also play a positive role in this process [67, 68]. Apart from these forthright antecedents that may have a direct effect, organizations may need to pay special attention to other indirect factors, such as employees’ willingness and voluntary effort to develop creative and innovative solutions [16]. Individual innovative behavior is positively associated with a dispositional tendency to engage in and enjoy thinking [69].

In 1977, Hurt developed a 20-item Likert-type scale for global innovativeness, which is defined as the willingness to change. Consumer innovativeness, defined as the proclivity to purchase new products more frequently and quickly than others, is a more limited or narrow concept than global innovativeness [60]. One of the earliest self-reported consumer innovativeness scales was developed by Goldsmith in 1991. With only six items measured on a 7-point agree-disagree scale, it is simple, reliable, and adaptable scale that can be employed across domains [70]. Employees’ innovativeness is discussed and measured, considering their innovative work behavior (IWB). IWB scales are Likert-type self-, peer-, and supervisor-report scales. Likert-type self-report scales are commonly used and have three distinct advantages: (1) they are simple and inexpensive to administer, (2) they can measure innovativeness across a variety of contexts, and (3) they have a high level of reliability [71]. Depending on the context, peer report scales are often deemed the most useful. For example, the peer report scale is very appealing to interdependent teams where employees do not have frequent contact with managers or supervisors and where peers are likely to be able to observe their colleagues’ work behavior regularly [69]. An objective measure can also provide assurance of the validity of the scale. One of the options could be to measure the individual’s total number of patents filed divided by the number of years they have worked for the company [4]. Another approach is to use both self-reports and supervisor reports, which Janssen believes is ideal for three reasons: first, employees can provide a more concise report on their innovative work behavior than their supervisor because they understand his/her contextual intentions and motives; second, IWB is discretionary, and the observer’s assessment of it is subjective; and third, supervisors may overlook many genuine innovative employee behaviors in favor of capturing only those intended to impress them [62].

The scales discussed thus far have been criticized for failing to address the multidimensionality of innovativeness, and some effort has gone into developing scales that can capture both creative and implementation dimensions [72, 73]. Furthermore, Lukes et al. attempted to capture all dimensions of innovativeness (exploration, generation, championing, and implementation of ideas) by developing and validating the innovative behavior inventory (IBI) and innovation support inventory (ISI) [74].

## Data and methods

### Data

The data for this study were retrieved on November 10, 2021, from the Clarivate Analytics Web of Science (WOS) Core Collection database. Other data sources such as Scopus are available, but the Web of Science allows data to be downloaded in an easy-to-use format for data analysis and mining techniques. The database is one of the most well-known indexing platforms and contains a diverse collection of multidisciplinary scientific studies [75]. It is frequently used and has been proven reliable in bibliometric studies and analyses [76]. For research on well-being, we used "well*being" as a search query. The asterisk is used to include articles that use alternative spellings of "well-being," such as "wellbeing." We used this query because the word "well-being" is an umbrella term that covers a range of concepts such as happiness, wellness, and life satisfaction. This is a multidimensional concept that includes subjective, objective, and social components. To research innovativeness, we used "innovativeness" as a search query. We used this query because we were more interested in investigating employees’ "ability to innovate" or "innovative behavior." In addition, we attempted to use "innovation" as a search query, but the results were too broad (more than 340,000 publications) because the word "innovation" is a generic term used in many different contexts unrelated to our research objective. We retrieved all articles (and their reference lists) that included these queries in either the title, abstract, or keywords. A total of 39,854 publications published between 1972 and 2021 were found using "well*being" and 7,093 were published between 1967 and 2021 for "innovativeness," representing all publications available until the retrieval day. We included all categories and document types (e.g., book chapters, editorial materials, and conference proceedings articles) to thoroughly investigate all possible research domains related to well-being and innovativeness.

We used broad queries and independently reviewed two research fields: well-being and innovativeness. When we used the combined query (“well*being AND innovativeness"), only a few articles (17 articles) were retrieved. Unexpectedly, most of these articles did not discuss the relationship between well-being and innovativeness, except for one study that investigated how personality traits are related to individual innovativeness and life satisfaction [77]. Using a broad search query also represents a challenge as retrieved a large number of publications (46,947 articles in total). However, bibliometric methods and data mining techniques allowed us to focus on the most relevant publications and assisted in navigating through a large number of documents.

### Methods

This study overcame four challenges in framing the relationship between employee well-being and innovativeness. First, we had to identify the dominant topics in well-being research; this step is essential for identifying related constructs and domains. Using the direct citation method, we built a citation network in which each article (represented as a node) was linked to any article in its list of references. As a cleaning step, only highly connected articles were chosen to construct the network, whereas disconnected nodes (not cited, with no citation, or weakly connected) were neglected. In a citation network, the disconnected articles are usually those that incidentally mention the query keywords but do not belong to the corpus of research, given that they do not cite any other paper on the topic. Based on the distribution of citations among articles, the network was grouped into research clusters using the modularity maximization algorithm (Louvain). Because papers rarely cite irrelative papers, the clusters are formed by articles that cite each other, assuming that they are on the same topic. We decided on the cluster’s dominant topic by reading the most cited articles and investigating their most-publishing journals, top authors, and most-occurring keywords. Thus, we were able to identify clusters that mainly discussed employee well-being, to which we collectively refer as "*the main cluster*." We also used the same method to produce sub-clusters of major clusters when we needed to investigate the sub-topics within. We used the same methods to identify the main topics in innovativeness research. This method is suitable for overviewing research fields and identifying main research topics and trends. It was used to explore the scientific research landscape for topics such as sustainability [78] and creativity [79]. It can also be effective in detecting and identifying new research topics and emerging research trends [80, 81]. Previous research by Shibata et al. compared different approaches to detecting research trends and found that direct citation is the best approach [82]. Second, within well-being research, we need to identify any constructs or factors associated with employee well-being (the main cluster). We achieved this by utilizing a term-based semantic similarity measure (cosine similarity) to determine the level of similarity between the main cluster (employee well-being) and other clusters within the well-being research. To calculate the cosine similarity value, we turn each cluster into term vectors and then calculate the cosine of the angle between these vectors. We used Eq (1) to calculate the cosine similarity value between clusters, such as between clusters 1 (C1) and 2 (C2) [83].


$$\cos(\theta_{C1,C2})=\frac{C1∙C2}{\left\|C1\|*\|C2\right\|}=\frac{\sum_{i}^{k}C1i\times C2i}{\sqrt{\sum_{i}^{k}{C1}^{2}}\times \sqrt{\sum_{i}^{k}{C1}^{2}}}\;0\leq \cos\left(\theta_{C1,C2}\right)\leq 1$$ (1)

The cosine similarity value of two identical documents (zero degrees between them) equals one. As the angle between the cluster’s vectors increases, the cosine similarity value decreases, with higher values indicating greater semantic similarity between them. The benefits of using the cosine similarity measure include its simplicity and the ability to work with texts of different sizes and indicate context, synonyms, and semantic similarity between texts. Third, we need to identify the clusters within innovativeness research that share a high level of semantic similarity with employee well-being (the main cluster). These clusters potentially represent innovativeness-related constructs and factors related to employee well-being. To achieve this, we calculated the cosine similarity between the main cluster and all other clusters and sub-clusters within innovativeness research. Finally, we constructed the framework and determined its components. We only considered clusters or sub-clusters (in both well-being and innovativeness research) that fulfilled the following inclusion criteria:

1. The cluster can be assigned to one of the following three levels: individual, organizational, and market, or can function as a link between them.
2. The cluster can be considered a construct or a factor that can influence or be influenced by employee well-being or innovativeness.
3. Evidence to support and explain its link to employee well-being or innovativeness (or its related constructs and factors) can be found in the literature within the cluster.

**Fig 1:**
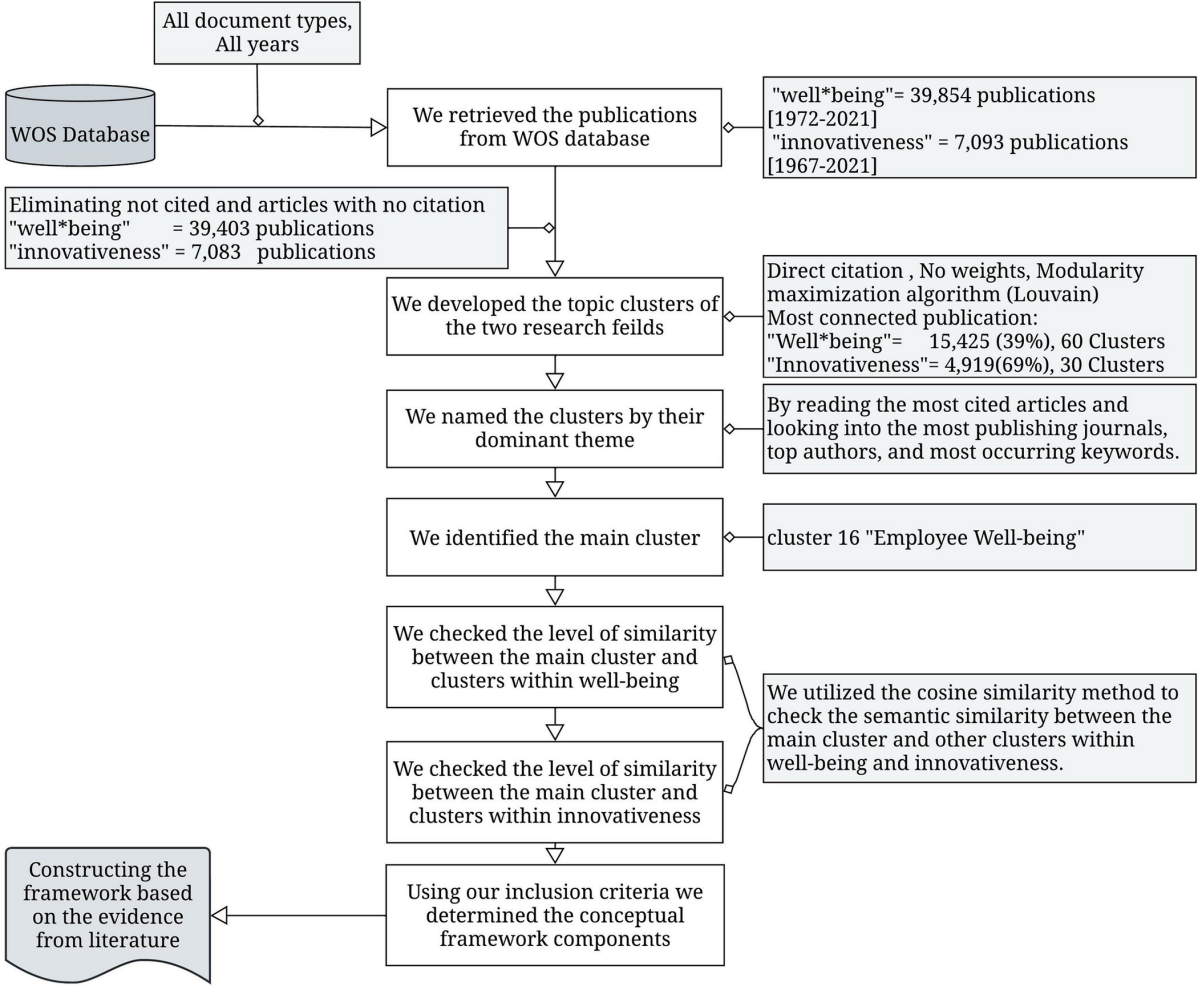
Flow chart summarizing the steps and methods we utilized to build our conceptual framework.

We only considered clusters that shared a high semantic similarity with the main cluster (with a cosine similarity value equal to or greater than the third quartile value (Q3) of the value distribution, which was selected as the threshold to indicate a potential association). However, to validate the effectiveness of this choice, we investigated the clusters that shared a similarity value below Q3, and we could not find any construct or factor that could be linked to employee well-being or innovativeness. The dynamics between the framework components and the direction of the relationship were determined and configured by investigating and reading the articles found within the clusters that represent the framework components. Fig 1 summarizes the steps and methods we used to construct our conceptual framework.

## Results

### Well-being

The citation network contained 29,140 links and 60 clusters. The large number of clusters suggests that well-being has been studied from different perspectives and discussed across various research fields. "Health" was the most frequently used keyword, and the average year of publication for all publications in the network was 2016. Table 2 shows the dominant themes, average publication year, top three journals, and the number of articles for the top 24 clusters (with more than 250 publications) in well-being research, which accounted for 78% of the total number of publications chosen as nodes.

**Table 2:** The top 24 clusters of well-being research named using the dominant theme with the most important quantitative data (number of articles, average publication year, top three journals, and number of articles in each journal) until 2021.

ID	Cluster Name	Articles (Percentage)	Ave. Year	Top 3 Journals	Articles
1	Green and natural environments	1,106 (7.17%)	2017.2	International Journal of Environmental Research and Public Health (IJERPH)	84
				Health & Place	64
				Landscape and Urban Planning	50
2	Family well-being	889 (5.76%)	2015.1	Journal of Marriage and Family	76
				Children and Youth Services Review	67
				Journal of Family Issues	39
3	Individual differences	778 (5.04%)	2017.2	Mindfulness	32
				Frontiers in Psychology	30
				IJERPH	21
4	Well-being interventions	760 (4.93%)	2016.8	Frontiers in Psychology	38
				Ageing & Society	30
				IJERPH	22
5	Ecosystems	693 (4.49%)	2016.6	Ecosystem Services	55
				Sustainability Basel	30
				marine policy	27
6	Child well-being	649 (4.21%)	2015.9	Children and Youth Services Review	31
				Child Indicators Research	28
				BMC Public Health	16
7	Subjective well-being	574 (3.72%)	2016.0	Social Indicators Research	45
				Journal of Happiness Studies	29
				Ageing & Society	14
8	Heritability and age	549 (3.56%)	2016.3	Ageing & Society	18
				Journal of Happiness Studies	16
				Social Indicators Research	14
9	Medical staff well-being	544 (3.53%)	2017.7	IJERPH	26
				International Review of Psychiatry	16
				BMC Medical Education	15
10	Personal Well-being Index (PWI)	541 (3.51%)	2014.5	Social Indicators Research	57
				Journal of Happiness Studies	34
				IJERPH	14
11	Aboriginal well-being (Australia)	535 (3.47%)	2016.4	IJERPH	32
				BMC Public Health	25
				The Australian and New Zealand Journal of Public Health (ANZJPH)	17
12	National mental health (Australian national surveys)	441 (2.86%)	2014.3	ANZJPH	67
				Journal of Affective Disorders	16
				BMC Public Health	14
13	Adolescent well-being	437 (2.83%)	2017.3	IJERPH	21
				PLOS One	19
				BMC Public Health	17
14	Refugee and migration	408 2.65%)	2016.8	BMC Public Health	15
				IJERPH	14
				Social Science & Medicine	13
15	Transportation and mobility	405 (2.63%)	2016.2	Ageing & Society	27
				IJERPH	22
				Journal of Transport & Health	18
16	Employee well-being	398 (2.58%)	2015.9	IJERPH	11
				The International Journal of Human Resource Management (IJHRM)	7
				Social Science & Medicine	7
17	Midwives and nurses	346 (2.24%)	2016.6	IJHRM	16
				Women and Birth	11
				Midwifery	10
18	Consumer satisfaction (Tourism)	326 (2.11%)	2015.7	Annals of Tourism Research	9
				Tourism Management	9
				Appetite	8
19	Internet and social media	316 (2.05%)	2017	Computers in Human Behavior	19
				IJERPH	15
				BMC Public Health	13
20	Pregnancy and childbirth	300 (1.94%)	2014.9	BMC Pregnancy and Childbirth	25
				An International Journal of Obstetrics & Gynecology (BJOG)	15
				BMC Public Health	13
21	Capability well-being	288 (1.87%)	2014.6	Social Science & Medicine	20
				Social Indicators Research	15
				Quality of Life Research	13
22	Animals	285 (1.85%)	2018.1	Anthrozoos	38
				Animals (Basel)	20
				Veterinary Record	18
23	COVID-19 pandemic	282 (1.83%)	2018.8	IJERPH	21
				Frontiers in Psychology	14
				PLOS One	10
24	Quality of work environment	251 (1.63%)	2015.9	IJERPH	10
				Sustainability Basel	7
				International Journal of Nursing Studies	7
25	Others: 36 clusters	3324 (21.55%)	-	-	-

Cluster 1 has the most articles (1,106 articles) and the dominant theme is green and natural environments. It discusses topics such as the impact of physical activities in natural environments on physical and mental well-being [84, 85], how blue spaces such as oceans impact health and well-being [86], their therapeutic potential [87], and the impact of green spaces on mental well-being [88] and stress levels [89]. Cluster 2 focuses on family well-being, with a particular emphasis on fragile families. Reichman’s paper (2001) is the most cited paper in this cluster as well as in all clusters on well-being (457 within-cluster citations). It discusses the definition of fragile family and is the first study to provide a thorough description of the research methodology and sampling strategies on the topic [90]. Cluster 3 examines individual differences in emotion regulation strategies and their relationship with personal and others’ well-being, with most studies focusing on the teacher–student relationship. Cluster 4 looks at how people use social and leisure interventions (music, singing, art, and other social prescriptions) and how these affect their well-being. Most of these studies focus on elderly and dementia patients. Cluster 5 focuses on fisheries and discusses social well-being and ecosystems. Cluster 6 primarily addresses children’s well-being in two settings: schools and foster care. Most of the papers in Cluster 7 deal with subjective well-being at the national level (country or continent). The paper with the most citations in this cluster investigated happiness in the United Kingdom and United States over time. One of the key findings is that happiness levels in the United States have decreased over the last quarter century, whereas life satisfaction in the United Kingdom remains relatively constant [91]. According to the authors, money and income also play a role in people’s evaluation of their well-being. Cluster 8 (heritability and age) focuses on the relationship between well-being and heritability, genetic influences, and non-shared environmental factors. One meta-analysis examines 30 twin family studies of well-being and concludes that the estimates of heritability in these studies show considerable variation, thus making it difficult to arrive at a conclusion [92]. In terms of its relationship with age, studies show that it has a U-shaped relationship [26] and that well-being boosts health and longevity [93]. The well-being of medical workers (trainees, physicians, residents, and medical students) is discussed in Cluster 9. The cluster discusses workers’ struggles with burnout, which is a major concern among medical professionals [94]. Researchers have also discussed interventions that could help prevent or reduce burnout [95]. Personal well-being indices (PWI) are defined, developed, and measured in Cluster 10. Most of the studies examine Australian well-being indices [42], with 37% of the articles (202 papers) published in Australia. Other studies examine PWI in other countries, such as China [96] and Algeria [97].

Small clusters with less than 250 publications were grouped as others (Table 2). The small number of publications may indicate that the topic is overlooked or contains research gaps that researchers should address. Some of these small clusters have clear dominant themes. For example, "family caregiving" is the main topic of Cluster 25 because most publications discuss the well-being of caregivers and care recipients (e.g., the elderly, people with psychosis, and dementia). In addition, the well-being of "athletes" is discussed in Cluster 33. In the next subsection, we discuss the clusters related to our research objectives in more detail.

### Innovativeness

A total of 4,919 out of 7,083 publications were included as nodes (69%) in the citation network, resulting in 21,854 links and 30 clusters. The average publication year for the entire set of publications was 2014. The most cited paper was an article by Ritu Agarwal and Jayesh Prasad published in 1998 entitled "Conceptual and Operational Definition of Personal Innovativeness in the Domain of Information Technology," which received 534 within-cluster citations. Table 3 shows the dominant theme, average publication year, top three journals, and the number of articles for the top ten clusters. The top 10 clusters accounted for 96% of all publications chosen as nodes.

**Table 3:** The top 10 clusters of innovativeness research named using the dominant theme with the most important quantitative data (number of articles, average publication year, top three journals, and number of articles in each journal) until 2021.

ID	Cluster Name	Articles (Percentage)	Ave. year	Top 3 Journals	Articles
1	Market orientation	1,070 (21.8%)	2014.4	Journal of Business Research	31
				Industrial Marketing Management	29
				Procedia—Social and Behavioral Sciences	20
2	Personal innovativeness	966 (19.6%)	2015.7	Computers in Human Behavior	43
				Telematics and Informatics	23
				Behavior & Information Technology	21
3	Product innovativeness	780 (15.9%)	2013.4	Journal of Product Innovation Management	104
				Industrial Marketing Management	28
				Journal of Business Research	22
4	Consumer innovativeness	724 (14.7%)	2013.4	Journal of Business Research	29
				Journal of Product Innovation Management	17
				Psychology & Marketing	14
5	Entrepreneurial orientation	637 (12.9%)	2016.1	Journal of Business Research	27
				Small Business Economics	17
				Journal of Small Business Management	14
6	Individual innovativeness	184 (3.7%)	2012.9	Procedia Social and Behavioral Sciences	6
				Sustainability Basel	5
				Hacettepe University Journal of Education	4
7	Brand innovativeness	122 (2.5%)	2017	Sustainability Basel	8
				International Journal of Hospitality Management	6
				European Journal of Marketing	6
8	Forest	93 (1.9%)	2011	Forest Policy and Economics	9
				Forest Products Journal	7
				Canadian Journal of Forest Research	6
9	Spatial innovativeness	82 (1.7%)	2015.7	Studies of the Industrial Geography Commission of the Polish Geographical Society	7
				Oeconomia Copernicana	4
				European Planning Studies	3
10	Policy innovativeness	76 (1.5%)	2012.3	Technology Analysis and Strategic Management	5
				Policy Studies Journal	4
				Research Policy	3
11	Others:	185	-	-	-
		3.8%			

Cluster 1 was the largest, accounting for approximately 21.8% of the total publications (1,070 articles), with market orientation being the most prevalent theme. The cluster focuses on the definition and conceptualization of market orientation [98], market orientation antecedents and their impact on business performance [99], market orientation’s relationship with innovativeness [100], and its relationship to customer satisfaction [101] and employee job satisfaction [102]. Owing to its size, this cluster covers a wide range of topics. We created sub-clusters using the same clustering method to successfully navigate our search through these topics. Fourteen sub-clusters were created. Organizational performance, learning orientation, small and medium enterprises (SMEs), leadership, environmental turbulence, organizational innovativeness, supply chain, energy, knowledge management, and ethical organizations were the top 10 sub-cluster’s dominant themes. The remaining sub-clusters were all small clusters with less than 30 publications. We discuss some of these sub-clusters in more detail in the following sections because they are relevant to our research goals.

Personal innovativeness is the dominant theme in the second cluster. This cluster’s most cited paper is by Agarwal, which is also the most cited among all the clusters [103]. To make the process of adopting new information technology more understandable, the authors developed a new model to define the construct of personal innovativeness in the domain of information technology, arguing that individual perceptions of new information technology are moderated by personal innovativeness. The cluster delves into the definition of personal innovativeness (defined by Agarwal as "*an individual’s willingness to try out any new information technology*") [103], as well as adoption models for various technologies, such as Internet shopping [104] and Online payment [105]. In addition, the influence of user psychological factors [106] and the antecedents and consequences of user perceptions of technology adoption [107] have received some attention. The dominant theme in Cluster 3 was product innovativeness: the authors focus on the relationship between product innovativeness (market familiarity, technological familiarity, marketing fit, technological fit, and new marketing activities) and product selection [108], performance [109], and success [110]. Personal innovativeness and how it may be affected by environmental turbulence are also discussed [111, 112]. Consumer innovativeness is the most prevalent theme in Cluster 4. The concept was first introduced and defined by Midgley and Dowling in 1978 as "*the tendency to buy new products more often and more quickly than other people*." Since then, its concepts, measurements, and validation have been thoroughly discussed [113, 114]. Because the concept of consumer innovativeness varies depending on the individual, empirical research discussing its relationship with personal characteristics and adoption behavior has been undertaken [115].

Entrepreneurial orientation is the focus of Cluster 5. Lumpkin’s 2001 article was the most cited in this cluster. Specifically, he discusses two dimensions of entrepreneurial orientation (proactiveness and competitive aggressiveness) and their relationship to firm performance, arguing that the former is positively related to performance, while the latter is negatively related [116]. As aforementioned, Lumpkin (1996) proposes five dimensions of entrepreneurial orientation—autonomy, innovativeness, risk-taking, proactiveness, and competitive aggressiveness—and discusses how these dimensions relate to performance [117]. The rest of the articles discuss these dimensions and their links to performance in different contexts, such as SMEs [118] and family businesses [119], as well as in specific industries, such as hospitality [120]. Cluster 6 focuses on individual innovativeness. Turkey is the country with the most publications in this cluster, with 30 publications out of 184. The topics it overs include the motivational antecedents of individual innovativeness in the workplace (innovative behavior in some studies) [121] and individual innovativeness measurement and validation [122]. The most common keyword in this cluster is teachers, indicating that many studies were conducted in a school context [123, 124]. However, individual innovativeness has also been discussed in other settings, such as hospitals (nurses) [125] and workplaces, where employee well-being has been identified as critical [17].

The dominant theme in Cluster 7 is brand innovativeness. The cluster focuses on the concept and operationalization of consumer perceptions of brand innovativeness [126] as well as how this perception affects brand loyalty [127] and attitudes toward new services [128]. Consumer perceptions of brand innovativeness are essential, as are those of firm innovativeness, because can influence consumer attitudes and behavioral intentions, which are moderated by gender and age [129, 130]. Cluster 8 looks at innovativeness in the forest sector [131], the forest product industry [132], and methods for measuring it [133]. Forest landowners were also discussed in terms of their innovativeness, antecedents, and the services they provide [134–136]. The next cluster examines innovativeness from a regional perspective. The most cited paper in this cluster introduces the concept of national innovative capacity, defined as "*a country’s ability to produce and commercialize a long-term flow of innovative technology*." The authors create a framework and use it as a guide to empirically investigate variations in innovation intensity at the national level [137]. The regional factors of innovativeness have been investigated in countries such as Germany [138], China [139], and Poland [140]. The final cluster we discuss is Cluster 10, which focuses on policy innovativeness at the state level ("state" is the most frequently used keyword in this cluster). For example, Boehmke uses event history analysis (EHA) to examine states’ willingness to adopt new policies sooner or later than in other states [141]. Nicholson-Crotty also creates a scale to assess a state’s collective policy innovation, claiming that he has successfully addressed internal and external validity issues previously encountered by other researchers [142].

**Fig 2:**
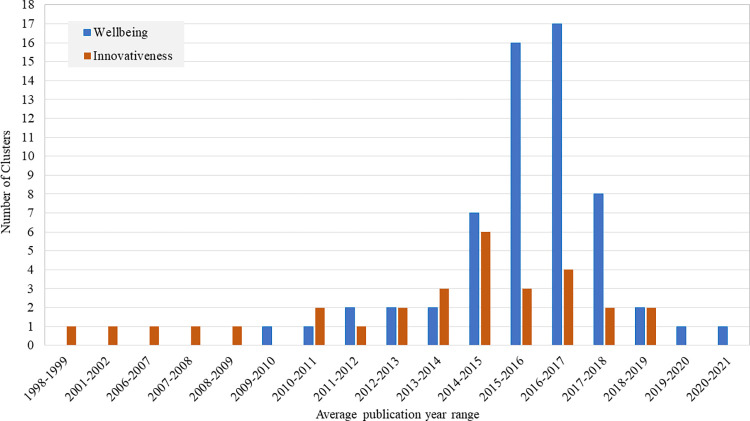
Average publication year range and numbers of clusters in each range for well-being and innovativeness research.

The remaining clusters were relatively small, with 45 articles or fewer. We grouped them as "Others" in Table 3. Owing to the small number of publications, it can be challenging to identify the dominant theme for these clusters. However, we recognized some prevalent topics. For instance, Cluster 11 (45 articles) primarily discusses e-commerce with a focus on cloud computing, the drivers and impediments surrounding its adoption, and the intention to continue using it [143, 144]. Animal innovativeness is widely discussed in Cluster 12, with birds being the most frequently discussed topic. Cluster 14 discusses local innovation ecosystems and how; for example, their structure may affect organizational adaptation capabilities [145]. Urban innovation (Cluster 16), start-ups (Cluster 17), innovation support (Cluster 18), drug development (Cluster 19), and business incubators (Cluster 20) are among the most discussed topics. Although Cluster 21 contains only four articles, its dominant topic is vital—the articles discussed innovativeness as a source of societal progress and enhancement of well-being [146]. One of the articles by Binder points out that these measures might be beneficial in innovative change, considering that society’s subjective well-being allows for an exhaustive assessment of the effects of innovativeness [147]. The average publication year range and number of clusters in each range (for all well-being and innovativeness clusters) are shown in Fig 2. The earliest average publication year range for innovativeness was 1998–1999, while that for well-being research was 2009–2010. The data suggest that the discussion of innovativeness in the literature precedes that of well-being. However, the earliest retrieved publication on well-being (in German) was by Mockel in 1939, entitled "Exercises of Health-and Well-being Institutions in the Four-Year Plan and During the War Economy." This article did not appear in our analysis because the WOS database indicated that it did not contain any references and had never been cited. Havens published the first paper on innovativeness (in English) in 1965 entitled "Increasing the Effectiveness of Predicting Innovativeness." Therefore, while the discussion on well-being may have begun prior to that on innovativeness, early publications on well-being must have been neglected because they were not among the most connected nodes.

The topics of clusters with an average publication year after 2016.8 (the third quartile of the distribution of the average publication years of all clusters) were considered emerging topics for well-being research. By contrast, topics of clusters with a publication year earlier than 2012.5 (the minimum value of the distribution of the average publication years of all clusters) were considered dying or saturated. Maternal well-being (Cluster 60), grief and social support (Cluster 56), and COVID-19 (Cluster 23) are the top three youngest emerging topics. On the other hand, four research topics were either dying or saturated: physical activity among the elderly (Cluster 47), obstetrics and gynecology (Cluster 48), injuries (Cluster 50), and fetal well-being (Cluster 35). Using the same logic as innovativeness research, we identified seven emerging research topics: entrepreneurial orientation (Cluster 5), brand innovativeness (Cluster 7), and clusters number 14, 19, 25, 27, and 30. In addition, there were two dying or saturated research topics: Clusters 21 and 26. All clusters mentioned by number contained only between three and seven articles, thus making their status as research topics debatable.

### Semantic similarity between clusters

**Fig 3:**
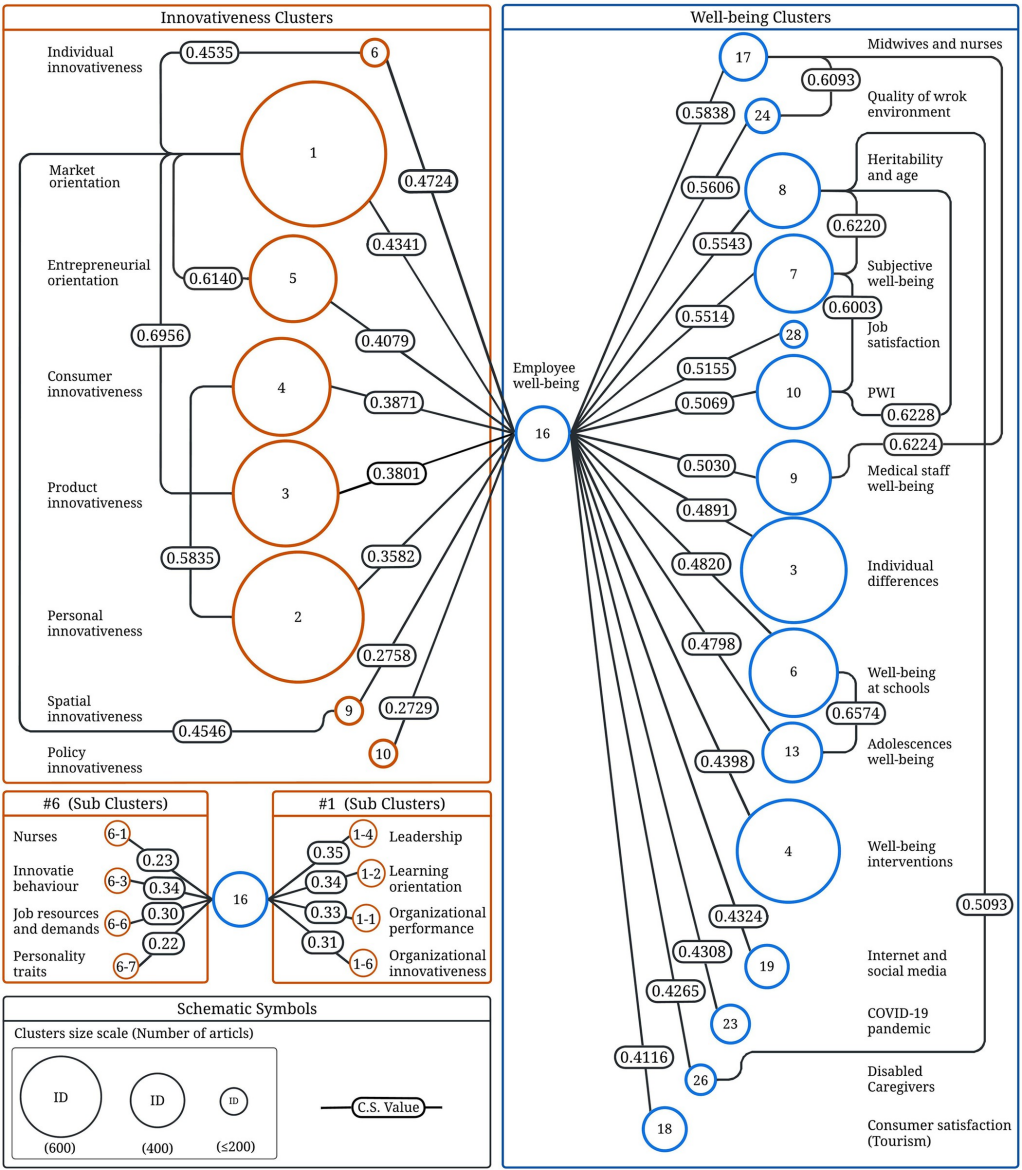
Schematic showing all clusters that share a cosine similarity value (with employee well-being) above the thresholds (third quartile). Relative clusters size and high cosine similarity values between clusters are also depicted.

As mentioned in the Methods section, we only considered the clusters that share a high semantic similarity with the main cluster (with a cosine similarity value equal to or greater than the third quartile value (Q3)). Using this threshold, eight clusters from the top 10 clusters in innovativeness research were identified (the top 10 clusters contained 96% of the total publications). The remaining clusters were 7 (brand innovativeness) and 8 (forest), both of which are outside the scope of this study. Some clusters in innovativeness contain many articles (e.g., cluster 1: market orientation) or have a multilayered relationship with employee well-being (e.g., cluster 6: individual innovativeness). We created sub-clusters for them and calculated their cosine similarity values with the main cluster to solve this problem. We extracted and sorted all clusters within the determined threshold, from which we identified 15 clusters within well-being and 16 clusters and sub-clusters within innovativeness. Fig 3 depicts the cosine similarity values between the identified clusters and the main cluster (employee well-being), with their relative size. The cosine similarity values between the clusters within each research field are also shown.

The semantic similarity measures seem to represent the relevance between clusters. For example, among the well-being clusters, Clusters 9 (medical staff) and 17 (midwives and nurses) shared the highest cosine similarity value (CS value = 0.6224), which can be explained by examining the dominant themes of these clusters. Both clusters discuss topics related to the well-being of employees working in the medical field. Moreover, they both share a high similarity with Cluster 24 (work environment quality), which focuses on the relationship between workers’ well-being and work environment qualities and issues such as bullying, job load, and burnout, all of which are common challenges for medical field workers. Clusters 7 (subjective well-being), 8 (heritability and ageing), and 10 (the Personal Well-being Index) also had high similarity values, which are due to the fact that the Personal Well-being Index is considered a national-level subjective well-being measure. Similarly, age and heritability are recurring topics that have been widely discussed in the subjective well-being literature. Among the innovativeness clusters, clusters 1 (market orientation) and 3 (product innovativeness) share the highest cosine similarity (CS = 0.6956). Product innovativeness is generally measured and assessed in two dimensions: the use of products new to the business and the market, with market orientation found to benefit both [148]. There is also a high similarity between the market orientation and entrepreneurial orientation clusters (CS = 0.614). Market orientation has a positive relationship with firm performance, both on its own and in certain aspects of entrepreneurial orientation. In addition, the relationship between market orientation and performance is moderated by two dimensions of entrepreneurial orientation: innovativeness and proactiveness [149]. Personal and consumer innovation clusters also shared a high cosine similarity value (CS = 0.5835) as these two are highly associated. Consumer innovativeness is the purchase or consumption of a new product or technology, whereas personal innovativeness is an individual’s willingness to experiment with and try new technologies [60, 103]. The smallest cluster (individual innovativeness) shares the highest cosine similarity value with the main cluster (CS = 0.4724), while Cluster 10 (policy innovativeness) has the lowest value (CS = 0.2729).

A remarkable finding in Fig 3 is that the main cluster (employee well-being) is the sole cluster bridging the two research fields under investigation (i.e., well-being and innovativeness). This does not mean that employee well-being is the sole factor in well-being and innovativeness, but that employee well-being works as a mediator between well-being and innovativeness. In the next section, we organize the clusters that fulfil our inclusion criteria into a multilevel conceptual framework. We then discuss the direct and indirect links that bridge the three levels supported by studies within these clusters.

## Discussion

As demonstrated in the previous section, eight clusters related to well-being and 13 related to inventiveness met our inclusion criteria. Seven innovativeness-related clusters were sub-clusters derived from Clusters 1 and 6. This section introduces the conceptual framework and discusses its theoretical and practical implications. It also describes the limitations and themes that future research may address.

### Conceptual framework

**Fig 4:**
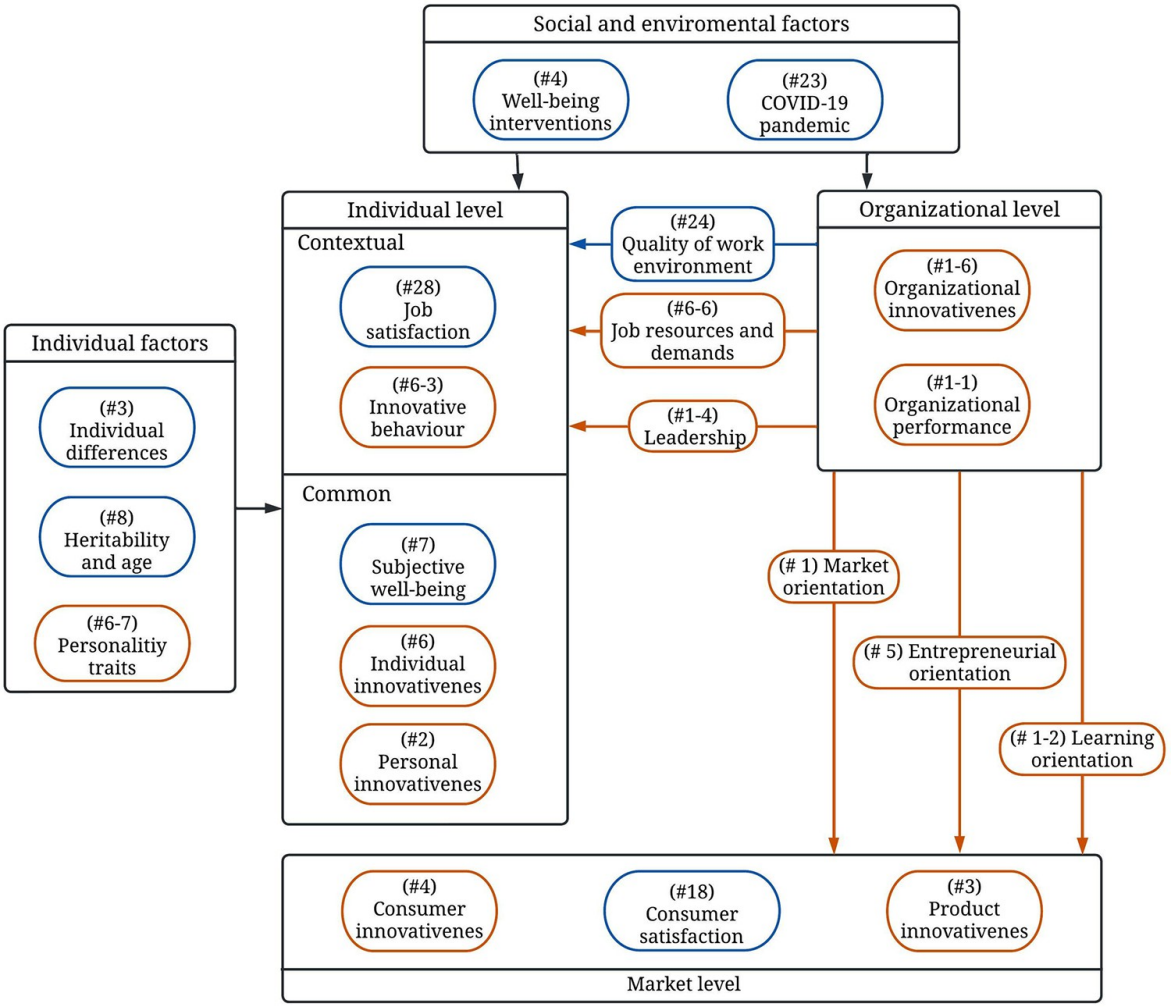
Multi-level conceptual framework depicting the relationship between employee well-being and innovativeness and other related constructs.

The multilevel conceptual framework is developed by assigning the clusters that fulfilled our selection criteria to one of the three levels (individual, organizational, and market) or can be considered as a link that connects these three levels. Clusters that influence any of the levels (e.g., social and environmental factors) are also depicted in the framework. The relationships between the framework components were decided and investigated by reviewing the articles within these clusters. We assigned any components related to individual well-being or innovativeness at the individual level, which was divided into two parts: contextual and common. The common part contains constructs with less consideration for the organizational context and the contextual part factors for the employee context. The organizational level contains components related to organizations such as organizational performance. For the links between the organizational and individual levels, we included factors that can be controlled or altered by organizations and affect the people working there. We placed components related to the market or consumers at the market level. Accordingly, we positioned any factor, activity, or process by organizations directed toward the market or consumers between the market and organizational levels. Fig 4 shows the conceptual framework components with their cluster or sub-cluster number. The components in blue are clusters related to well-being, while the brown clusters are related to innovativeness research. Social and environmental factors, as well as individual factors, are also depicted.

In the next four subsections, we review the literature and introduce some studies that explain and justify the links between framework components. We will start from the individual level, proceed through the organizational and market levels, and finally discuss the other factors depicted in the framework.

**Individual level.** Subjective well-being and job satisfaction are constructs of well-being at this level. Along with psychological well-being and workplace well-being (job satisfaction and work-related affects), subjective well-being is considered one of the main constructs of employee well-being [40]. Subjective well-being, an individual’s evaluation of his/her own life, consists of three primary constructs: life satisfaction and positive and negative dispositional affects. Regarding its relationship with innovativeness, Dolan et al. found a correlation between creativity and subjective well-being in the general population, where originality and imagination increased in line with higher life evaluations of individuals [5]. In the working context, the opposite relationship is also true, that is, working in a creative environment and being involved in innovative behavior gives employees a sense of control that can influence their work and lead to well-being [17]. Moreover, employee well-being is strongly connected to organizational commitment, which is considered a well-being construct, along with job satisfaction and personal satisfaction [150]. The authors confirmed a strong and positive link between employee well-being and job performance, with job satisfaction and organizational commitment being the most influential variables. Therefore, employees and employers need to be aware of and build resilience against factors that can negatively influence this level’s well-being constructs (i.e., subjective well-being and job satisfaction). For example, in their meta-analysis, Faragher et al. discussed three factors that have strong relationships with job satisfaction: burnout, self-esteem, and depression [151]. To reduce work-related stress, job support is essential as its absence can have a negative impact on mental health [152].

This level depicts three innovative components: individual innovativeness, innovative behavior, and personal innovativeness. Individual innovativeness is the ability to perceive and react to new ideas, inventions, or ways of doing things, and the ability to generate ideas and accept challenges [71]. This construct is connected to innovative behavior because a person with high individual innovativeness is more likely to engage in innovative behavior in an organizational context. This innovativeness construct is critical because organizations rely on employees’ innovative behavior to solve problems and overcome everyday work challenges. To understand why employees engage in innovative behavior in the workplace, Yuan et al. examined how innovative behavior is explained by expectations of such behavior (expected positive performance outcomes, image risks, and image gains) [121]. Their findings demonstrated that all three expectation outcomes had a significant impact on innovative behavior. Notably, employees may avoid engaging in innovative behavior because they fear being negatively perceived by others. Expectations are shaped by contextual and individual factors, such as job satisfaction, organizational support for innovation, and relationship quality with supervisors. On the other hand, personal innovativeness is related to an individual’s tendency to try new products or technologies; thus, it is closely connected to consumer innovativeness [103]. Knowledge about consumers’ needs is vital for employees to innovate because their knowledge or ability to find solutions must be coupled with their knowledge of customers’ needs. Schweisfurth et al. investigated this aspect with the participation of 864 employees and found that solution and need absorptive capacity are distinct and positively associated with employee innovativeness [153]. To absorb solutions or customer needs knowledge, both of which can be external knowledge, employees need to reach beyond their organization’s boundaries. However, this may expose employees to conflicting demands. Schweisfurth et al. also studied how identity conflict can affect employees’ innovative behavior. According to their study, identity conflict can decrease job satisfaction if employees are strongly identified with both organization and users. Moreover, identity conflict is negatively related to innovative behavior through job satisfaction [154]. In addition, when we consider temporary settings (where people come together for a short time to work on a particular problem), innovative behavior is significantly affected by experimentation, creative self-efficacy, and perceived task orientation [155].

#### Organizational level

This level contains one construct related to innovativeness—organizational innovativeness—and one construct that can be considered a consequence of the other related constructs: organizational performance. The most cited article in Clusters 1–6 (organizational innovativeness) criticizes previous studies for conceptualizing organizational innovativeness as a unidimensional construct [156] and reports three shortcomings: 1) focusing on one or a few types of innovation, 2) assessing innovativeness based on the number of adopted innovations, and 3) evaluating innovativeness at only one point in time. It proposes the need to consider the consistency, mean time of innovation adoption, and number of innovation adoptions over time and concludes that there is a substantive relationship between organizational factors and organizational innovativeness. Some of the main factors explaining organizational innovativeness are related to organizational contexts, such as organizational size and slack, which are positively linked [157]. As expected, organizational innovativeness is closely linked to performance. This relationship between organizational innovativeness and performance can be direct [156] but organizational innovativeness can also mediate the relationship between performance and other factors, such as market orientation [158], knowledge management [159, 160], learning orientation [100, 161], transformational leadership [162, 163], and entrepreneurial orientation [164]. Organizational innovativeness also strongly mediates the effects of organizational culture and leadership on performance [165].

Employee well-being has a positive relationship with organizational innovativeness [17], and evidence supporting that employee well-being is a predictor of organizational performance has been widely presented and discussed [166, 167]. Quality of the work environment, job resources and demands, and leadership represent organizational factors that impact individuals. Next, we discuss the bridging factors between the individual and organizational levels.

*Quality of work environment.* Respectful personal relationships, flexible work, supportive management, and good communication all contribute to a healthy work environment [168]. Organizational culture is also an essential factor comprising employees’ personal and shared views, attitudes, and assumptions about their company, which subsequently influence their actions [169]. Moreover, organizational culture can greatly influence employees’ overall well-being [168], especially job commitment and satisfaction [170]. It also moderates the relationship between employees’ inner motives and creative performance [171], with highly innovative cultures being more conducive to transforming creative ideas into actual innovation [172]. However, work environment qualities, such as bullying, create a poor environment that can adversely affect employees’ psychological well-being, which is also negatively related to concentration [173]. Employee well-being and innovativeness are influenced not only by emotional and psychological factors but also by physical aspects of the working environment. For example, Gilchrist investigated the relationship between employees’ well-being and their use of green spaces and window views and concluded that well-being can be predicted by the amount of time spent in green spaces at work [174]. Well-being also mediates the relationship between the physical environment and employee commitment [175]. Additionally, architecture and interiors can influence individuals’ well-being [176]. An empirical study found that even light levels and colors can affect workers’ moods [177]. A positive mood is proposed as a precursor to creative ideas, which can positively influence and support creativity [178, 179]. Workers can also be more creative in environments designed to be intellectually and perceptually stimulating [180].

*Job resources and demands.* An empirical study involving 1,525 registered nurses found that self-reported job demands and job strain were risk factors for employee well-being [181]. A positive impact on well-being can be achieved by gaining structural and social resources, which can be enhanced by allowing employees to change and redesign job aspects (tasks, interpersonal relationships, and the cognitive stance toward their work) [182]. Creativity and innovativeness are impeded by stress and burnout caused by long-term work demands, but flourish when employees have sufficient job resources [183, 184]. Another interesting argument by Huhtala suggests that innovativeness itself can be a job demand when considering the challenges embedded in the concept and searching for novel solutions to problems, but can also be a job resource when its costly consequences are well managed [16].

*Leadership.* Employees’ well-being and innovativeness are influenced by leadership, which is typically channeled through aspects related to leadership or leadership style. Skakon et al. reviewed 30 years of leadership literature and found that leader and employee stress and well-being are connected, suggesting that leader stress is transferable [185]. The negative behavior of the stressed leader can explain this adverse effect. On the other hand, leaders’ positive behavior can enhance employees’ active well-being [186]. Another factor is leader humility, which is found, through transformational leadership, to have a favorable effect on employee positive affect, job satisfaction [187], and team innovativeness [188]. Mindfulness has also been found to have a positive impact on employee well-being [189] and IWB [190]. Leaders’ mindfulness means having a non-judgmental awareness and concern about events and surrounding situations internally (thoughts and feelings), externally (physical and social), and being open to new experiences [191, 192]. One of the interesting factors of a leader is his/her positive humor, which has a positive effect on employee well-being [193] and creativity through work engagement [194]. Authentic leadership has also been found to positively impact employee well-being [195] and innovativeness [67].

#### Market level

Consumer innovativeness refers to the tendency to purchase a product or service before others [60]. This construct, along with personal innovativeness, is important for predicting consumer loyalty and behavior. Consumer loyalty is positively associated with perceived firm innovativeness [129]. The market level also contains another vital component—product innovativeness—defined as the utilization of new products that are new to both the company and the market. Product innovativeness is associated with product success in the market, but organizations need to be cautious about generalizing this for every case. Szymanski et al. argue that despite a small-to-medium correlation between product innovativeness and performance, the correlation could become very strong when various measurement and context factors are considered [196]. Calantone et al. also warn businesses against relying solely on product innovativeness for success, arguing that doing so may undermine customer familiarity, which could negatively impact a product’s success [110]. The authors also stress the importance of using distribution channels to offset customers’ apprehensions about unfamiliar products. According to another study by Danneels et al., market and technological familiarity, marketing and technological fit, and new marketing activities are five dimensions of product innovativeness that have unique relationships with market decisions and product performance [108]. Another factor to consider when it comes to product innovativeness is how quickly organizations want to get their products to the market. When customers are involved in developing new products (as an information resource or a codeveloper), Fang suggests that they may have to choose between product innovativeness and speed of market introduction [197]. These findings emphasize the importance of consumer and market knowledge for organizations owing to their critical role in market decisions. Market knowledge can influence product performance (success or failure) [198]. The ability to successfully manage knowledge (internal or external) and have a market-, learning-, and entrepreneurial-oriented culture is positively associated with organizational innovativeness and, as a result, organizational performance [100, 159, 161, 199].

The only well-being-related component identified at this level was consumer satisfaction, which is closely linked to product performance. It is defined as an evaluation based on comparing product performance after purchase with consumer expectations prior to purchasing a product or service [200]. This cluster (consumer satisfaction) focuses mainly on the tourism sector, and the most cited paper in it discusses how hedonic and eudemonic well-being can be derived and enhanced through tourist activities and experiences [201]. Interestingly, consumer satisfaction is also related to employee job satisfaction. Homburg et al. find a positive relationship between salespeople’s job satisfaction and customer satisfaction, particularly for products with high innovativeness. This relationship becomes more potent when salespeople need to interact more frequently with customers [202]. In addition to product innovativeness, Ordanini et al. argue that service innovativeness is **positively** related to firm performance (revenue and profit growth) and employee well-being [203].

*Market orientation*. The influence of market orientation on organizational innovativeness and performance and its relationship to product innovativeness and consumer satisfaction is widely discussed in the literature. However, their relationship with employee job satisfaction is uncommon. Siguaw et al. investigate this relationship by surveying 46 dairy cattle milk cooperatives in Indonesia and find that organizational learning and high job satisfaction can lead to high market orientation [102]. The opposite direction relationship is also discussed, where high market orientation can lead to high job satisfaction [102, 204, 205], prevent job dissatisfaction [206], increase trust in management [204], and enhance organizational commitment [204].

*Entrepreneurial orientation*. As previously mentioned, Lumpkin (1996) argues that there are five dimensions of entrepreneurial orientation: autonomy, innovativeness, risk-taking, proactiveness, and competitive aggressiveness. Iqbal et al. discuss the association between these two dimensions and organizational performance, finding that proactiveness is positively related but competitive aggressiveness tends to be poorly associated with performance [164]. Another study discusses this relationship during the embryonic stage of firm growth [199], but their findings suggest that competitive aggressiveness and autonomy have no influence. In contrast, proactiveness and innovativeness positively influence business performance, while risk-taking has a negative effect.

*Learning orientation*. Based on 25 in-depth field interviews with R&D vice presidents and a literature review, Calantone et al. introduce four learning orientation components: commitment to learning, shared vision, open-mindedness, and organizational knowledge sharing [207]. This study argues that learning orientation is essential for innovation and that organizations need to fully understand their environment, including customers, competitors, and emerging technologies. In addition to the positive relationship between learning orientation and organizational performance, organizational innovativeness mediates this relationship.

#### Other factors

We identified five other factors that are related to individuals’ well-being and innovativeness: individual differences, well-being interventions, heritability and age, COVID-19 pandemic, and personality traits. Three out of the five identified factors can be considered individual factors because they vary from one person to another: individual differences, heritability and age, and personality traits. The remaining factors can be categorized as social and environmental factors that can affect employee well-being or innovativeness or their related constructs at any level: well-being interventions and the COVID-19 pandemic.

*Individual differences*. Five studies conducted by Gross (the most cited paper in this cluster) examine how individual differences and people’s use of emotion regulation strategies, such as reappraisal and suppression, affect their well-being and social relationships [208]. One of these studies concludes that reappraisal is positively associated with well-being, whereas suppression is negatively related. Researchers have also investigated other potential individual differences, such as the levels of mindfulness [209], self-compassion [210], emotional intelligence [211], and depressive symptoms [212]. Most of the studies in this cluster have focused on the school context and the relationship between students and teachers.

*Heritability and age.* Subjective well-being and health are closely linked to age. Diener et al. argue that based on experimental research on humans and animals, the evidence that subjective well-being influences health and longevity is credible. However, the claim that subjective well-being extends the lives of people with diseases is debatable [93]. Regarding the genetic nature of well-being, Bartels et al. conclude in their meta-analysis study based on 30 twin family studies on well-being, that due to the considerable variation in heritability estimates in these studies (0%–64%), it is difficult to confirm the genetic influence on well-being [92]. We could not find any studies within this cluster that had more than 64% variance due to genetic factors [213, 214], which means that heritability varies across populations, subgroups, contexts, and constructs. However, when exploring stable SWB levels, heritability variance can reach approximately 80% [215].

*Personality traits*. Personality traits have been proposed as antecedents for two constructs: individual inventiveness and life satisfaction, where some traits are positively associated—extraversion, agreeableness, conscientiousness, and openness to experience—and one trait has negative association: neuroticism [77]. The same study also argues that there is a positive association between individual innovativeness and perception of quality of life. Another study examines the effects of entrepreneurs’ personality traits on firm innovativeness; using a sample of 2,574 small- and medium-scale manufacturing enterprises, they conclude that three personality traits are positively related to entrepreneurs’ innovativeness: extraversion, conscientiousness, and openness to experience, whereas neuroticism is negatively related [216]. This study also argues that agreeableness has an insignificant influence on entrepreneurial innovativeness and firm innovation performance.

*Well-being interventions*. Well-being interventions can be utilized at an individual level for the general population (e.g., the elderly and people with dementia) and in the organizational context (i.e., employees). Social and leisure interventions (e.g., group activities, singing, and music) are widely discussed in this cluster. For example, a systematic review targeting interventions for older people concludes that approximately 80% of participatory interventions produce a positive effect. Yoga, as a well-being intervention, has received considerable attention. Indeed, most of the studies in cluster 28 (job satisfaction), claim it positively impacts well-being [217] and increases resilience to stress [218]. Other interventions, such as cultural events or activities, have been studied in an organizational context. Tuisku et al. conclude that cultural events for employees might enhance their occupational well-being. Furthermore, participation in such activities positively affects work engagement and seems to mediate the innovative work climate measured by support for new ideas [219]. Choral singing has also been studied in an organizational context where singers, compared to non-singers, demonstrated higher engagement and organizational commitment levels and reported a positive change in their psychosocial work environment [220].

*COVID-19 pandemic*. The average publication year for this cluster was 2018.8, which makes it the youngest cluster among those with more than 100 articles (41 clusters). The pandemic has significantly affected people, organizations, and almost all countries’ economies. A study in the UK examining the trajectory of people’s mental health during lockdown surveyed 3,077 adults and found that the pandemic had negatively affected their mental health and well-being, with alarming increases in suicidal thoughts [221]. One month after the pandemic, some employees stopped working, others started working from home, and others continued working at offices. According to a study from China, those who stopped working reported worse mental health, physical health, and distress; in some cities, the severity of COVID-19 predicted life satisfaction [222]. Physical activity, tragic optimism, gratitude, and social support were found to be beneficial for protecting well-being during the pandemic [223].

### Practical and theoretical implications

Our framework presents three essential organization-related factors regarding employees’ well-being and innovativeness: quality of work environment, job resources and demands, and leadership. Managers should support their employees by increasing job resources, reducing job demands and stress, and creating a high-quality work environment in which employees can experience more positive effects. Leaders need to lead by example, deliver high leadership qualities, be humble, and exhibit positive attitudes. To enhance and influence employees’ innovative behavior, they also need to build a stimulating environment, encourage new ideas and novel endeavors, grant their employees some degree of control over their tasks, and allow them to learn and improve their skills. At the same time, employees must work towards solving their customers’ problems and addressing their needs through innovative products and services. Our framework depicts three constructs regarding the market and consumers: market, entrepreneurial, and learning orientation. To provide value and increase their organizations’ innovativeness and improve their performance, employees need to understand and have deep knowledge of the market condition and be aware of their customers’ level of satisfaction. Market knowledge is vital for product innovativeness and market decisions and can influence product performance (success and failure). Having a learning-oriented culture and efficiently managing this knowledge (internal or external) is essential for organizational innovativeness and, consequently, its performance. Organizations need to be entrepreneurially oriented and have a relatively high risk tolerance, affording employees the opportunity to experiment with products and ideas. Organizations can also benefit from involving customers in new product development to boost innovativeness. Understanding consumer innovativeness is vital for predicting loyalty and behavior toward new products and services introduced in the market.

Our analysis confirms the findings of Dolan and Honkaniemi, who affirmed the association between subjective well-being and innovativeness [5, 17]. Since subjective well-being is an individual’s evaluation of his/her own life, managers in organizations could regard it as a personal issue. However, since it is one of the employee well-being constructs (besides workplace well-being and psychological well-being) and, thus, one of the important antecedents of organizational innovativeness and performance, managers need to pay more attention to this construct. In addition to employee well-being, managers should put more effort into how employees’ innovativeness can and should be enhanced. Being involved in innovative activities and behaviors can enhance employees’ well-being (subjective, workplace, and psychological) through giving them a sense of control over their tasks; moreover, we can expect more creative work from employees when they have a high level of well-being and when they are praised and encouraged for their innovative behavior.

The link between subjective well-being and innovativeness is critical for governments. Policy innovativeness is the dominant theme of Cluster 10, the research on which discusses policy innovativeness at the state level. Based on our analysis, it is plausible that policy innovativeness is linked to policymakers’ well-being and innovativeness. Although it can be challenging to prove a causal relationship between subjective well-being and innovativeness, higher quality of life evaluations in the general population are linked to greater originality and imagination. In other words, creativity and subjective well-being are correlated. Creativity is the first dimension of innovativeness (idea generation); this idea was explored by Binder, who highlights the implications of this relationship for the performance and progress of societies [147]. He also argues that theories about the dynamic nature of subjective well-being could allow for a comprehensive assessment of the effects of innovativeness on society. Accordingly, it may be prudent to place subjective well-being at the center of researchers and policymakers’ discussions regarding innovativeness and economic development. In addition, policymakers (at the state and national levels) need to be creative, redefine their priorities, and reform their processes to be more agile and efficient in addressing the critical and urgent issues and challenges that society may face. Addressing societal issues and problems will improve people’s well-being and have a positive effect on how they view and evaluate their lives.

**Fig 5:**
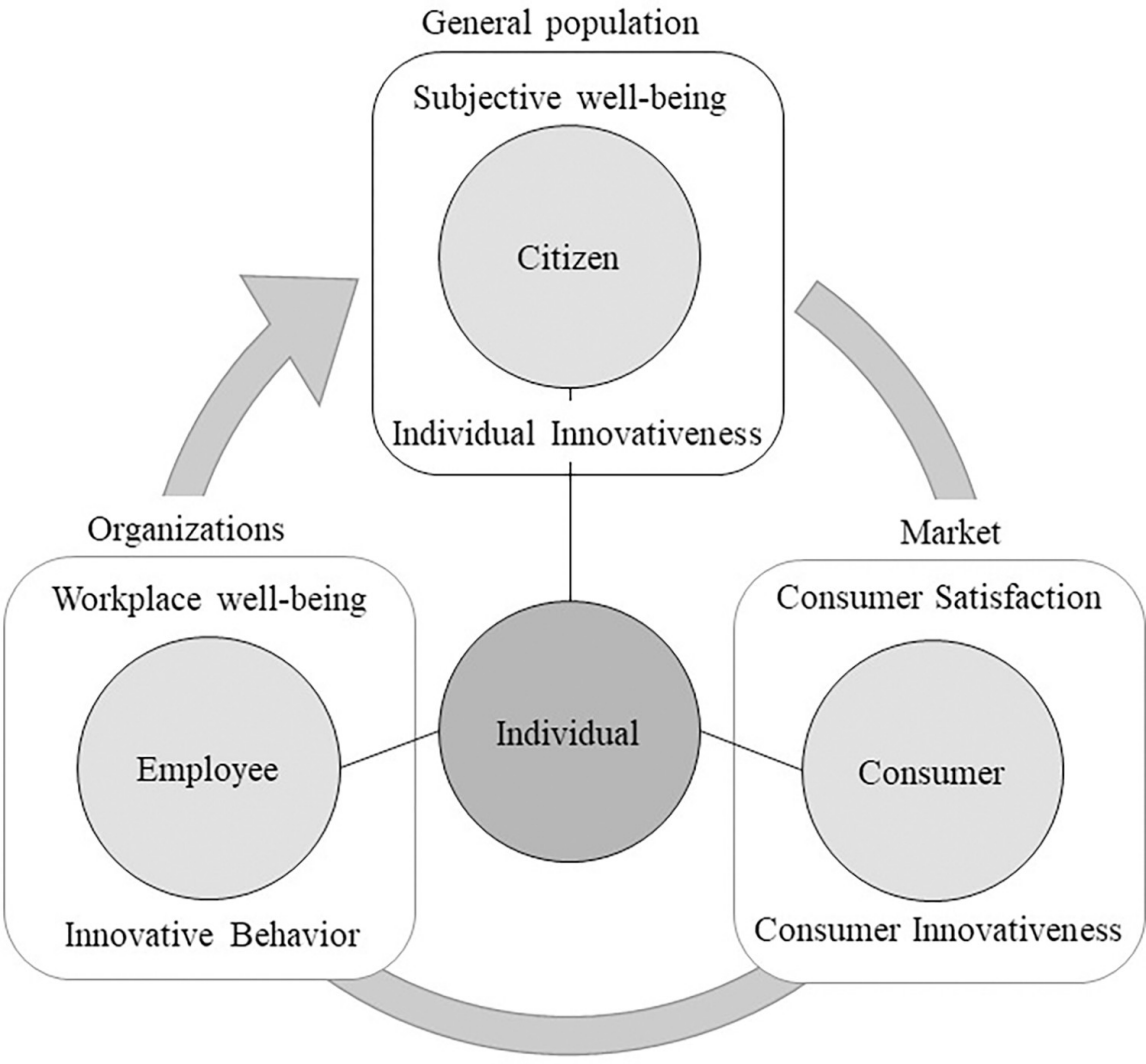
Abstraction showing contextual well-being and innovativeness constructs for an individual.

Our analysis also revealed the criticality of well-being and innovativeness in various contexts of our lives as they are fundamental to our ability to make positive contributions to society and to enjoy a satisfying existence. Everyone has various needs and responsibilities depending on the situation, but for every context (e.g., citizen, employee, consumer) well-being and innovativeness seem to be vital constructs that are associated with these needs and responsibilities (Fig 5).

An individual is not usually confined to a single context. For example, when it comes to the responsibilities and tasks associated with one’s position, a policymaker (also a citizen) is an employee and a consumer of goods and services. For each context, we can identify a construct related to well-being and innovativeness (e.g., job satisfaction and innovative employee behavior). While their role may vary, every individual needs to be innovative in solving problems and issues in a way that will improve both their own and others’ well-being. This, in turn, will increase their capacity to innovate. Social and environmental factors can affect the contextual constructs of well-being and innovativeness. These factors can be attributed to various reasons and can affect the constructs of a single context or many (cross-context factors). Changes in consumer demand and lifestyles, economic conditions, the emergence of new technologies, and health crises represent examples of such factors. The COVID-19 pandemic, for example, was a cross-contextual factor that influenced almost all contextual and non-contextual well-being and innovativeness constructs. This forced us to redefine some concepts (e.g., how we work and study) and look deeper into some fixed assumptions regarding the theoretical framework that influences our decision-making practices. Therefore, despite contextual situations, everyone needs to work together to enhance these constructs’ resilience against external disruption so that we can continue bringing value to our society and achieve a satisfying existence.

### Research prospects and future agenda

Having a broad, overarching view of the two research fields and exploring their landscapes can be helpful for many stakeholders, such as academics, organizational leaders, and policymakers. For academics, this could help expand our understanding of the topics under investigation and discover relationships and factors that might be difficult to recognize using traditional review methods. It could also help identify research gaps and overlooked research themes. Leaders and policymakers can utilize insights from such studies to direct future efforts, build effective strategies, and determine the best ways to allocate resources.

Based on our extensive literature review and insights from the conceptual framework, we propose three broad themes that future research may address. The first is related to measurement and covers the question of how employees’ well-being and innovativeness (innovative behavior) should be measured. Appropriate scales should consider the relationship between their related domains and constructs at three levels: individual, organizational, and market. Moreover, the scales must cover all construct dimensions (e.g., idea generation, promotion, and implementation) and have the flexibility to accommodate various job contexts (e.g., teleworking, part-time, site, and office). In addition, understanding how cultural differences across countries influence the development and formulation of measurement items is crucial.

The second theme is proactive and covers the question of how we can enhance employee well-being and innovativeness. This research theme can be compared to the previous theme in terms of construct dimensions, cultural differences, and job contexts. Focusing on one level might not be sufficient since factors related to other levels might have a more significant impact that could undermine our single-level efforts.

The last research theme is related to policies and broadly covers two aspects. The first aspect covers how to develop more rapid and accurate measures or indicators of society’s well-being and innovativeness, which can be critical for policymakers to determine the success or failure of policies under implementation. It also has economic benefits as it can assist in proper resource allocation and reduce unjustified government spending. The second aspect is related to policy innovativeness and centers on how we can speed up policy development and implementation, as well as make it more responsive to our rapidly changing world. Taking a long time to develop and implement a new policy can be disastrous, particularly when dealing with urgent and rapidly escalating situations and crises. Finally, it is important to note that studying or investigating research questions developed around these three themes would require interdisciplinary and multi-stakeholder approaches as the questions are complex and involve multiple actors.

### Limitations

Combining citation network analysis with data mining techniques for investigating relationships between constructs is an original approach and was found to be useful for our study. However, because our study is based on citation network analysis, we may have overlooked some new emerging research themes that have already begun to proliferate owing to the limited number of publications discussing these topics. Similarly, a newly published article will not appear among the most cited papers in the clusters. However, as mentioned in the Methods section, citation analysis is generally considered effective in detecting and identifying new research topics and emerging research trends. Another concern with our bibliometric analysis method is that the Web of Science core database is the only data source. Therefore, we cannot be sure that we included all relevant publications on well-being and innovativeness. However, the database is frequently used, has proven valuable for gaining broad perspectives on research fields, and is a reliable tool for bibliometric research analysis. Finally, the study focused only on the individual, organizational, and market levels. There might be other levels worth examining, but this was not addressed in this research.

## Conclusion

This study presents comprehensive qualitative and quantitative reviews of 49 and 54 years of well-being and innovativeness research, respectively. Moreover, considering all relevant constructs and factors at the individual, organizational, and market levels, we present a holistic overview of the relationship between employee well-being and innovativeness by proposing a conceptual framework comprising 21 components. These components consist of constructs, factors, and related consequences and can influence or be influenced by employee well-being and innovativeness (directly or indirectly) through different mediators. It also identifies other social, environmental, and individual factors that can affect some of these constructs. We discuss the relationships between these components, highlighting the multilevel nature of the relationship between employee well-being and innovativeness. Notably, the multilevel and interdisciplinary nature of the relationship needs to be considered when attempting to measure or enhance these constructs. The proposed framework could aid in developing a multilevel measurement of employee well-being and innovativeness and help direct future research efforts.

Our analysis shows that for an organization to enhance its employees’ well-being and innovativeness, it needs to increase job resources, lower its demands, create a high-quality working environment, and improve leadership competency. Market and consumer knowledge were found to be critical factors of employee job satisfaction and innovative behavior. Thus, managers should encourage employees to pursue knowledge that lies outside the immediate scope of their work and behind organizational barriers. Additionally, for a successful innovative endeavor, employees may need to take risks and be proactive and well-established norms and practices may have to be challenged. For organizations to realize the full potential of their employees and attain high organizational innovativeness and performance, an all-level integrated effort needs to be exerted. Such a concerted effort is critical to ensure consumer satisfaction, product innovativeness, and market performance. Finally, as employees are essential resources for any organization, companies need to enhance their employees’ well-being and innovativeness resilience. Managing resilience can be an efficient strategy that for organizations to deal with and learn from uncertainties and unexpected circumstances (e.g., COVID-19).
